# Therapy related myelodysplastic syndrome: a hematologic sequela of low dose methotrexate in rheumatoid arthritis

**DOI:** 10.1093/omcr/omaf253

**Published:** 2025-11-26

**Authors:** Ayushi Gupta, Venugopala D, Alam Nawaz, Vivek Hari

**Affiliations:** Department of General Medicine, Kasturba Medical College Mangalore, Manipal Academy of higher education, Manipal, 203, Light House Hill Road, Hampankatta, Mangaluru, Karnataka 575001, India; Department of General Medicine, Kasturba Medical College Mangalore, Manipal Academy of higher education, Manipal, 203, Light House Hill Road, Hampankatta, Mangaluru, Karnataka 575001, India; Department of General Medicine, Kasturba Medical College Mangalore, Manipal Academy of higher education, Manipal, 203, Light House Hill Road, Hampankatta, Mangaluru, Karnataka 575001, India; Department of General Medicine, Kasturba Medical College Mangalore, Manipal Academy of higher education, Manipal, 203, Light House Hill Road, Hampankatta, Mangaluru, Karnataka 575001, India

**Keywords:** therapy related myelodysplastic syndrome, low dose methotrexate, rheumatoid arthritis, pancytopenia, leucovorin, filgrastim

## Abstract

**Introduction:**

Methotrexate (MTX) is a key drug in rheumatoid arthritis (RA) management but can rarely cause life-threatening hematologic toxicity, including therapy-related myelodysplastic syndrome (t-MDS), even at low doses.

**Case Presentation:**

A 58-year-old male with RA on low-dose MTX (7.5 mg/week) presented with pancytopenia. Evaluation showed Hb 5.7 g/dL, TLC 1300/μL, and platelets 63 000/μL. Nutritional deficiencies, infections, and autoimmune flare were excluded. Bone marrow examination revealed dysplasia with 11% blasts and abnormal precursors, suggestive of t-MDS. MTX and leflunomide were discontinued. He received leucovorin, G-CSF (Granulocyte colony stimulating factor), and supportive care. Rapid hematologic recovery was observed.

**Conclusion:**

This case highlights the potential for low-dose MTX to cause t-MDS, which may be reversible if detected early. As serum MTX levels do not correlate with toxicity, regular blood count monitoring is essential. Prompt drug withdrawal and supportive therapy can lead to full recovery and prevent permanent marrow damage.

## Introduction

Rheumatoid arthritis (RA) is a chronic autoimmune disease primarily affecting synovial joints, but it can affect multiple organs in advanced stages. Methotrexate (MTX), remains the first-line Disease modifying anti rheumatic drug (DMARD) due to its effectiveness in controlling inflammation and halting disease progression [1]. Unlike well-documented high-dose MTX (HD-MTX) toxicity in oncology; hematologic toxicity from low-dose MTX (LD-MTX) in autoimmune diseases is underrecognized. LD-MTX can rarely lead to lymphomas and therapy-related myelodysplastic syndrome (t-MDS); manifesting as pancytopenias [2]. t-MDS has worse outcomes than de novo MDS and may result from MTX-induced folate pathway disruption, DNA damage, and clonal hematopoiesis. Risk factors include renal dysfunction, age, folate deficiency, and methylenetetrahydrofolate reductase (MTHFR) polymorphisms. We report a case of low-dose MTX-induced t-MDS in a 58-year-old RA patient.

## Case report

A 58-year-old male with a 10-year history of hypertension and RA presented with complaints of fever, cough, generalised weakness, and multiple joint pains since two weeks. He was on methotrexate (MTX) 7.5 mg once weekly, hydroxychloroquine (HCQ) 200 mg twice daily, leflunomide (LEF) 10 mg once daily, folic acid 5 mg once daily for RA and telmisartan for hypertension.

During one of the follow up visits with his physician, the patient was found to have developed pancytopenia (shown in Table 1 as Day −15).

**Table 1:** Baseline investigations before presentation to the hospital.

	Day − 15 (Before admission)
Haemoglobin (g/dL)	5.7
Haematocrit (%)	18.6
RBC Count (million/cumm)	1.9
TLC (cells/microL)	1300
Platelet count (cells/microL)	63 000

Methotrexate was stopped (on Day −15) due to pancytopenia, while leflunomide and HCQ were continued. He was referred to our hospital for further management.

On arrival, he appeared pale with bilateral knee joints and multiple metacarpophalangeal joints swollen and tender. No lymphadenopathy or palpable abdominal organomegaly. Other systems were within normal limits.

Laboratory investigations revealed pancytopenia (Table 2).

**Table 2:** Laboratory investigations on day 1 of admission at our hospital.

Haemoglobin	7.9 g/dl
White blood cell count	1210 cells/μl
Platelet count	70 000 cells/μl
Neutrophils	26%
Lymphocytes	65%
Monocytes	3%
Eosinophils	4%
Basophils	2%
ESR	140 mm/hr
Reticulocyte count	2.1%
LDH	608 IU/L
Peripheral smear examination	Leukoerythroblastic anaemia with 6% blast cells

Additional investigations were done to determine the underlying cause of pancytopenia (Table 3).

**Table 3:** Additional investigations on day 1 of admission at our hospital.

Vitamin B12	2000 pg/ml
Folic Acid	20 ng/ml
Procalcitonin	0.03 ng/ml
Blood culture	No Growth
Urine Culture	No Growth
HbsAg	Negative
Anti-HCV	Negative
HIV 1&2 Ag/Ab	Negative
EBV IgM	Negative
CMV IgM	Negative
Malaria by Peripheral Smear and rapid diagnostic kit	Negative
Dengue IgM	Negative
Leptospirosis IgM	Negative
Widal test	Negative
ANA	Negative

Vitamin B12 deficiency, sepsis, tropical fevers, and viral infections were ruled out, making secondary infection an unlikely cause of pancytopenia and arthritis flare.

Abdominal ultrasonography revealed mild fatty hepatomegaly, and spleen appeared normal.

In view of the persistent pancytopenia, a bone marrow examination was performed on day 2 of admission.

**Figure 1:**
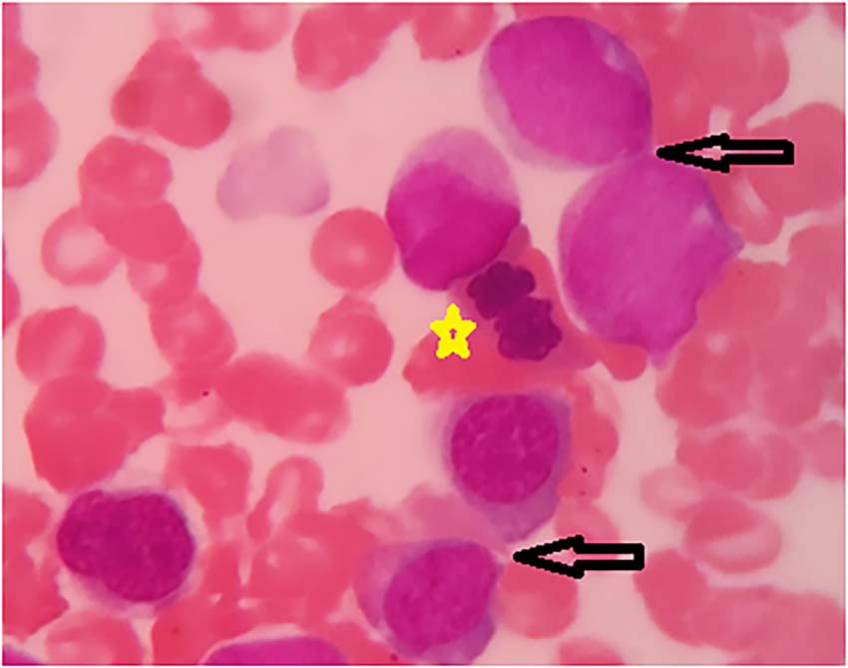
Bone marrow aspirate showing dyserythropoiesis (star) with megaloblastoid forms (arrows).

Bone marrow aspirate revealed dyserythropoiesis with megaloblastoid forms (Fig. 1).

**Figure 2:**
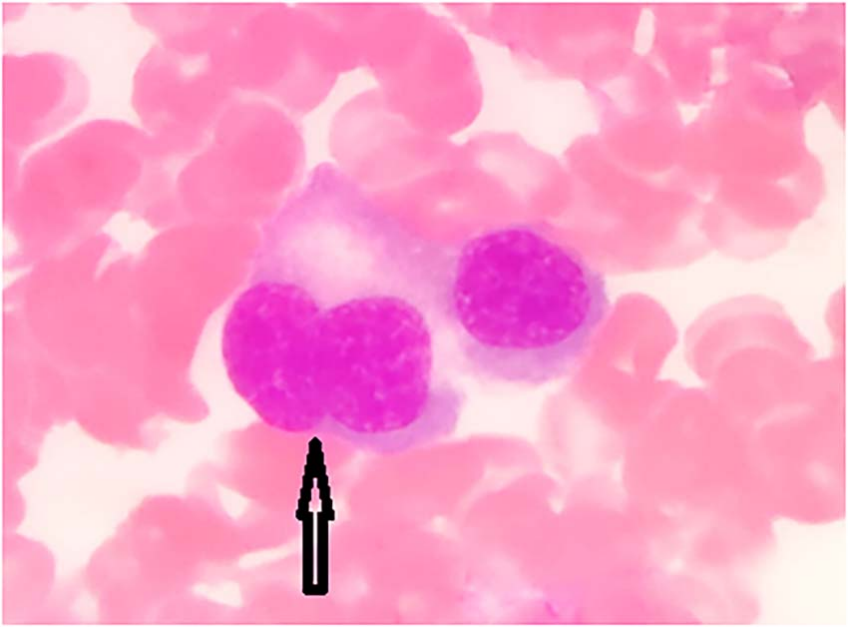
Multinucleated forms with nuclear budding (arrow).

**Figure 3:**
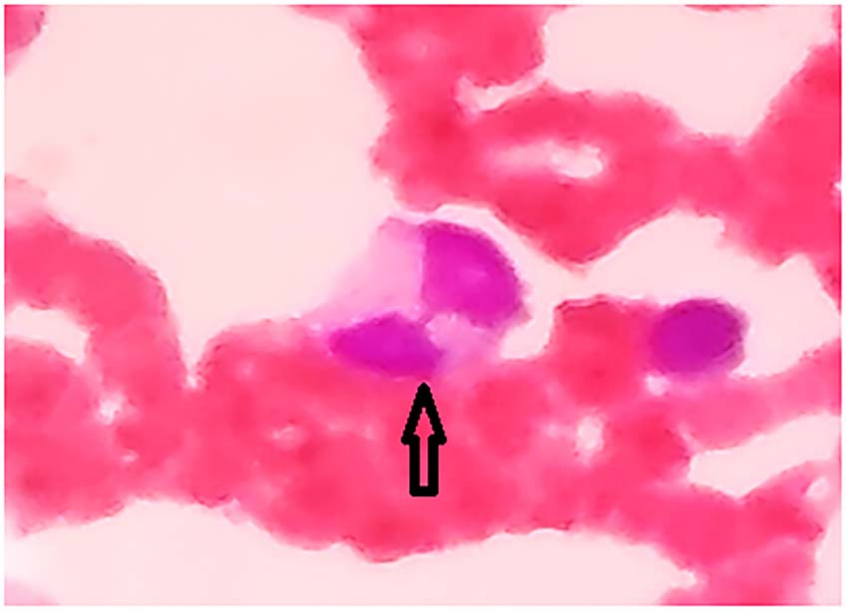
Multinucleated forms with nuclear bridging (arrow).

Along with multinucleated forms with nuclear bridging and nuclear budding (Figs. 2 and 3).

Granulopoiesis was decreased. Promyelocytes and 11% myeloblasts were also observed.

**Figure 4:**
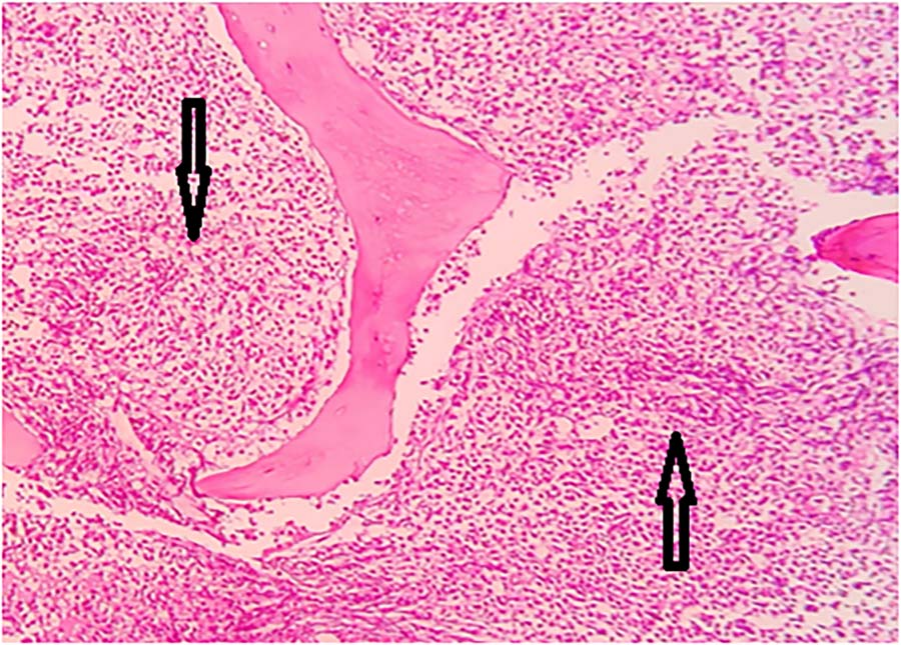
Hypercellular marrow with sheets of monotonous, medium sized cells- eosinophilic cytoplasm and prominent nucleoli.

Bone marrow biopsy was suggestive of hypercellular marrow with sheets of monotonous medium sized cells having pink cytoplasm, rounded nuclei and prominent nucleoli (Fig. 4).

**Figure 5:**
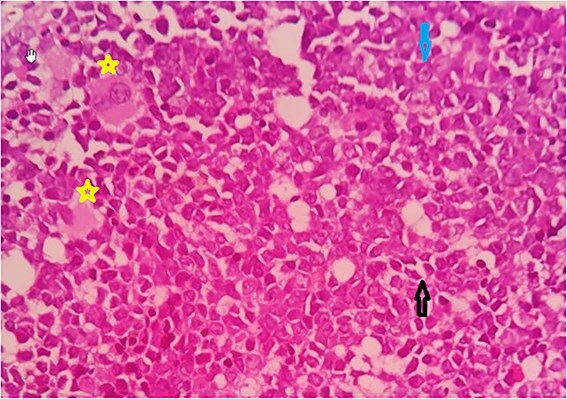
Mature and dysplastic megakaryocytes (star), plasma cells (arrow facing down), eosinophils, and immature myeloid precursors (arrow facing up).

Figure 5 shows mature and dysplastic megakaryocytes along with plasma cells, eosinophils, and immature myeloid precursors.

Histopathological findings were suggestive of Therapy induced Myelodysplastic Syndrome with 11% blast cells.

Flow cytometry and next-generation sequencing (NGS) were not done due to unavailability. Temporal association with methotrexate exposure and MDS development, along with bone marrow findings were highly suggestive of t-MDS over de novo.

Methotrexate withdrawal (from Day −15; prior to admission) was continued and Leflunomide was also withheld (from Day 1 of admission). He was managed with leucovorin (folinic acid) rescue therapy, IV hydration and G-CSF (granulocyte colony stimulating factor) injections.

The patient responded to treatment, and pancytopenia resolved. Follow up CBC values on day 4 showed improvement. (Table 4). Day 7 showed resolution of pancytopenia.

**Table 4:** Follow up CBC after stopping MTX (on day −15) and LEF (on day 1).

	Day − 15 (Before admission)	Day 1 (On admission)	Day 4 (After admission)	Day 7 (At discharge)
Haemoglobin (g/dL)	5.7	7.9	8.8	10.7
Haematocrit (%)	18.6	24.1	27	34.2
RBC Count (million/cumm)	1.9	1.83	2.68	5.1
TLC (cells/microL)	1300	1210	1740	5100
Platelet count (cells/microL)	63 000	70 000	90 000	1,20 000

The normalization of pancytopenia was utilized as a marker of Methotrexate toxicity resolution. Patient was discharged subsequently on Hydroxychloroquine.

## Discussion

About 37% of RA patients will discontinue MTX therapy due to complications. Low-dose toxicity remains largely underreported unlike high dose toxicity [2].

Methotrexate inhibits dihydrofolate reductase, causing folate deficiency which triggers DNA hypomethylation and proto-oncogene activation. t-MDS/AML often involve specific chromosomal deletions and genetic polymorphisms in MTHFR [3].

Though histology shows overlapping features; findings like hypocellular marrow, atypical megakaryocytes, and higher blast counts, favour t-MDS over de novo MDS. Unavailability of flow cytometry and NGS were limiting, however the temporal relationship of MTX use and the onset of myelodysplasia, marrow morphology, and resolution of pancytopenia following drug withdrawal, supported the diagnosis of t-MDS [4].

Paradoxical arthritis flare and raised ESR may stem from pro-inflammatory cytokine release from myeloid cells. Also, immune dysregulation in t-MDS may cause reactivation of RA [5].

There are no specific guidelines for management. Abrupt withdrawal of methotrexate, Leucovorin to reduce myelosuppression and Filgrastim (G-CSF) to aid neutrophil recovery are advised. Combined with leucovorin and MTX withdrawal; filgrastim shows faster, synergistic recovery [1, 6]. Our patient also showed rapid hematologic improvement.

LD-MTX toxicity is idiosyncratic and not dose-dependent, typically occurring despite normal serum drug concentrations [6]. Serum MTX were not checked in our patient due to limited resources and poor correlation between MTX levels and toxicity severity. Recovery from MTX-induced cytopenias usually occurs within 4–7 days following MTX withdrawal and initiation of leucovorin and/or G-CSF.

High-dose MTX requires serum level monitoring at 24,48 and 72 hours to guide leucovorin therapy. In contrast, as per the American College of Rheumatology, LD-MTX requires CBC, LFTs, renal tests and chest X ray at baseline and repeat at every 2–4 weeks during the first 3 months, every 8–12 weeks from 3–6 months, and every 12 weeks thereafter [7].

Leflunomide-related pancytopenia is rare. Hill et al. found only nine cases among 12 700 exposures, mostly with concurrent MTX [8]. Unlike reported LEF-induced marrow aplasia, our patient showed dysplasia and 11% myeloblasts, indicating MDS [9]. Given leflunomide’s prolonged half-life, cholestyramine washout is usually required. Hydroxychloroquine has minimal MDS association with only one reported case of MDS, lacking mechanistic backing [10]. Recovery after MTX withdrawal, without Leflunomide washout and without HCQ cessation, suggests MTX as the cause, with less likelihood of synergistic DMARD toxicity.

## Conclusion

Low-dose methotrexate toxicity is underrecognized and can cause therapy-related MDS especially in genetically susceptible individuals. Serum MTX levels don’t predict toxicity; regular CBC and organ function monitoring is key. Prompt MTX cessation, leucovorin, and G-CSF supported rapid hematologic recovery. Vigilance and early detection of pancytopenia is crucial, even with long-term low-dose MTX therapy.
